# Recruiters’ lived experiences in COVID-19 vaccine trials in eThekwini, KwaZulu-Natal

**DOI:** 10.4102/hsag.v30i0.3123

**Published:** 2025-11-19

**Authors:** Kumari Dewrance, Shenuka Singh

**Affiliations:** 1 School of Nursing and Public Health, College of Health Sciences, University of KwaZulu-Natal, Durban, South Africa; 2 HIV and Other Infectious Diseases Research Unit, South African Medical Research Council, Durban, South Africa; 3 Discipline of Dentistry, College of Health Sciences, University of KwaZulu-Natal, Durban, South Africa

**Keywords:** recruitment, COVID-19 vaccine trials, recruiters, perspectives, KwaZulu-Natal, South Africa, lived experiences

## Abstract

**Background:**

The coronavirus disease 2019 (COVID-19) pandemic led to widespread COVID-19 vaccine trials globally, with South Africa hosting many of these studies. Participant recruitment for these trials has been hindered by vaccine hesitancy, misinformation, distrust and pandemic-related restrictions.

**Aim:**

This study explored the experiences and perceptions of recruiters involved in COVID-19 vaccine trials in eThekwini, KwaZulu-Natal, focusing on recruitment facilitators, barriers and strategies.

**Setting:**

Six clinical trial sites in eThekwini, KwaZulu-Natal.

**Methods:**

Using a qualitative phenomenological approach within an interpretive paradigm, researchers conducted in-depth online interviews via Microsoft Teams with 14 recruiters selected through non-probability purposive sampling. Field notes supplemented the interviews, and thematic analysis with open coding identified key themes.

**Results:**

Analysis of recruiters’ lived experiences revealed four main themes: (1) motivations driving participation, (2) barriers faced in the field, (3) strategies used to engage communities and facilitate enrolment and (4) recruiters’ insights for improving future recruitment success.

**Conclusion:**

This study identified the difficulties recruiters faced in enrolling participants in COVID-19 vaccine trials in eThekwini, and the reasons why participants chose to take part. Insights from recruiters can inform strategies to strengthen participant recruitment in future clinical trials.

**Contribution:**

This research provides context-specific recommendations for improving recruitment during public health emergencies, with implications for future trials in similar settings.

## Introduction

In response to the global spread of coronavirus disease 2019 (COVID-19), the World Health Organization (WHO) declared the outbreak a pandemic on 11 March 2020 (WHO 2020). The severe acute respiratory syndrome coronavirus 2 (SARS-CoV-2) virus has since infected an estimated 760 million people worldwide, resulting in approximately 6.9 million confirmed deaths (WHO 2023). In response, developing COVID-19 vaccines became a top global research priority. By 30 April 2022, a total of 108 vaccine-related trials were conducted in Africa, with South Africa leading the way, having the highest number of trials at 58 (Wiysonge et al. 2022). For countries such as South Africa, conducting clinical trials is critical to generating local evidence on vaccine safety and efficacy.

Recruiting participants remains a major obstacle to clinical trial success and is a leading cause of trial discontinuation because of the inability to enrol the required number of participants. A systematic review of 65 COVID-19 trials in Germany showed that only 13.4% of the planned participants were recruited (Hirt et al. 2021). Vaccine hesitancy, misinformation, fear of side effects and conspiracy theories have further complicated efforts to recruit participants for COVID-19 vaccine trials (Abdelhafiz et al. 2021; Biesty et al. 2024). In South Africa, historical distrust and limited public engagement with researchers have contributed to hesitancy in clinical trial participation (ASSAf 2009). A study found that historical mistrust in South Africa because of past exploitation during apartheid has led to skepticism towards HIV vaccine trials, particularly in black, low-income communities (Thabethe et al. 2018). Despite these recruitment difficulties, South Africa’s strong clinical infrastructure, low litigation, and diverse health issues make it an attractive location for clinical trials (ASSAf 2009). High unemployment and limited access to medication often motivate clinical trial participation. The COVID-19 pandemic has further intensified recruitment difficulties for clinical trials, with lockdowns, movement restrictions, and social distancing measures reducing participant availability and accessibility regionally and globally (Sathian et al. 2020). Despite global reporting on these barriers, there remains a significant gap in the literature on the specific lived recruitment experiences of clinical trial recruiters of COVID-19 vaccine trials in South Africa’s eThekwini District, highlighting the need for focused research in this context.

A qualitative synthesis of studies from the United Kingdom and the United States identified key factors influencing COVID-19 vaccine trial recruitment, such as trust in safety, clear communication, demographic factors, incentives, accessibility, and personal motivations, while highlighting the need to address vaccine hesitancy and logistical barriers to ensure diverse participation (Biesty et al. 2024). In other global contexts, various unique barriers to participant recruitment have been reported. For instance, a study among young adults in China highlighted concerns over time commitments, hesitation around signing informed consent documents and perceived societal stigma related to COVID-19 as significant barriers to trial participation (Sun, Lin & Operario 2021). In the United States, researchers struggled to recruit diverse populations, particularly high-risk groups, older people and racial minorities slowing the progress of vaccine trials (Jaklevic 2020). Within Africa, a survey conducted in Uganda revealed that 54.6% of healthcare workers were reluctant to participate in COVID-19 vaccine trials because of safety concerns, fear of the virus, stigma, injections and time commitments (Kitonsa et al. 2021). Likewise, in India, community health workers showed reluctance to participate in trials because of a lack of trust in vaccines (Goel et al. 2022). These findings suggest that low willingness to vaccinate can significantly exacerbate recruitment difficulties, forming a significant barrier to successful trial implementation, an issue that is likely to affect recruitment efforts in South Africa as well. However, there is a paucity of published evidence on clinical trial recruiters’ lived experiences in dealing with these barriers to participant recruitment.

Additionally, there is limited evidence on the perspectives and experiences of COVID-19 clinical trial recruiters in KwaZulu-Natal. Therefore, understanding the unique barriers to participant recruitment within the South African context is critical to improving recruitment strategies and ensuring the success of local vaccine trials. This article emanated from a dissertation submitted for a master’s degree by the first author.

This qualitative phenomenological study aimed to describe and understand the lived experiences and perceptions of community-based recruiters involved in COVID-19 vaccine trial recruitment. The study’s objectives were to examine the facilitators and barriers encountered during recruitment and to explore recruiters’ strategies. By focusing on the eThekwini District, this study provided valuable data that can be used to improve recruitment efforts in both future COVID-19 vaccine trials and other clinical trials in similar settings. The findings can inform study design, recruitment planning, and implementation strategies for future trials, ensuring they are more inclusive and effective in engaging diverse populations.

## Research methods and design

### Study design

A qualitative phenomenological approach was chosen for this study to explore the lived experiences and perceptions of recruiters involved in recruiting participants for COVID-19 vaccine trials. This approach is particularly suited for understanding subjective experiences, as it allows the researcher to gain insight into how recruiters interpret their roles, difficulties recruiters face in enrolling participants and interactions within specific social and cultural contexts. While clinical research managers often rely on quantitative data to assess recruitment outcomes, a phenomenological approach, informed by the interpretative paradigm, offers a deeper understanding of recruitment’s complex, context-dependent nature. This approach helped gain insight to capture recruiters’ detailed insights and lived experiences, which cannot be fully represented by numerical data alone. Thus, it is well-suited to explore the difficulties recruiters faced in enrolling participants in marginalised communities, where social and cultural factors play a significant role in the recruitment process. The study employed a phenomenological approach, following Moustakas’ four-step methodology (1994) as adopted and summarised by (Greening 2019:88–92): ‘(1) defining the phenomenon of interest, the lived experiences of recruiters in COVID-19 vaccine trials, (2) bracketing the researcher’s assumptions to minimise bias, (3) conducting in-depth interviews (IDIs) to elicit recruiters’ perspectives on facilitators, barriers and recruitment strategies and (4) thematically analysing participants’ quotes and statements within the context of the clinical trial sites, communities and organisational environments’.

### Study setting

The study was conducted at six clinical research sites affiliated with two major research institutions in the eThekwini District, KwaZulu-Natal, South Africa. These sites represented diverse geographic and socio-economic contexts, including urban Durban Central, semi-rural Botha’s Hill, the densely populated suburb of Chatsworth, peri-urban Isipingo and Tongaat and mixed township and town neighbourhood in Verulam. All sites were actively recruiting participants for multiple COVID-19 vaccine trials, allowing the study to capture recruiters’ lived experiences across varied social, cultural and organisational environments. Collectively, the sites recruited participants from urban, peri-urban and township settings across eThekwini, applying the principle of maximum variation to capture a range of recruiters’ lived experiences and enhance the transferability of the findings. Recruiters participated in a variety of trials, including studies evaluating the safety and efficacy of protein-based vaccines among HIV-negative and HIV-positive participants, single-dose viral vector vaccines across multiple countries and booster dose strategies, including mix-and-match approaches for healthcare workers. Recruitment occurred across multiple sites, such as community health clinics, hospital outpatient departments, old-age homes and research facilities providing recruiters with exposure to diverse participant populations and contextual challenges. This study explored the lived experiences of recruiters involved in COVID-19 vaccine trials in the eThekwini district, South Africa. The aim was to gain an in-depth understanding of recruitment processes, including the number and types of trials recruiters were involved in, the sites where they recruited participants, their years of experience and their perceptions of participant motivations and recruitment difficulties and the strategies used to strengthen participant recruitment. The study focused on capturing personal, context-specific experiences, reflecting the operational, social and logistical realities recruiters faced during the pandemic.

### Study population and sampling strategy

The study population consisted of 14 fieldworkers and community recruiters from six clinical research sites of two research organisations in the eThekwini District, KwaZulu-Natal, whose primary role was recruiting participants for COVID-19 vaccine trials. Inclusion criteria required participants to have at least 3 months of experience in COVID-19 vaccine trial recruitment in the district, be willing and able to provide informed consent and be involved in either completed or ongoing COVID-19 vaccine trials.

The sampling strategy employed was non-probability purposive sampling, where the researcher deliberately selected 14 fieldworkers and community recruiters who were well-informed and could provide rich insights into participant recruitment for COVID-19 vaccine trials (Etikan 2016). The adequacy of the sample was assessed based on data quality and thematic saturation, not on the number of participants in the sample. Participants for this study were recruited through collaboration with community managers at both research organisations. The researcher held in-person and Microsoft Teams meetings with the participants to introduce the study, explain its voluntary nature and clearly communicate the eligibility criteria, study participation details and selection process. Interested recruiters contacted the researcher via email or telephone, and after confirming their interest, interviews were scheduled at mutually convenient times. All 14 recruiters who expressed interest were included in the study, with no refusals or dropouts during the interview process.

### Data collection

The researcher conducted interviews between February 2023 and May 2023. The interview technique used was conversational but purposeful, guided by semi-structured questions and enhanced through probing and reflective listening to gain depth.

The researcher had previous experience conducting qualitative interviews during a Postgraduate Diploma in Public Health and through the qualitative research methods module in the Master’s in Public Health programme. The training equipped the researcher with skills in building rapport, active listening and responsive probing to facilitate open dialogue. These techniques supported the depth and quality of the data collected. All interviews were conducted via Microsoft Teams, which automatically recorded the sessions with participants’ consent. The researcher cross-checked the transcripts against the Microsoft Teams recordings to ensure accuracy and consistency. Microsoft Teams does not always capture all accents clearly, so the researcher carefully re-listened to each recording to correct and refine the transcripts before analysis. The interviews took place in quiet, comfortable rooms at the clinical research site or in the participant’s home, with only the participant and researcher present. While the interviews were conducted online, they took place in these private, controlled environments to ensure comfort and confidentiality during the conversation.

The research instrument was an interview guide with open-ended questions designed to ensure comprehensive coverage of the topics. The guide began with background questions to build rapport and make participants feel comfortable. The main interview questions focused on the recruiter’s role, lived experience of difficulties faced in recruiting participants for COVID-19 vaccine trials, factors influencing participant decisions and strategies to improve recruitment.

Probing was used to encourage participants to clarify, elaborate or explain their responses, allowing for deeper insights. To deepen the interviews, the researcher used Gordon’s (2023) probing framework, which includes six types of probes: silent (allowing uninterrupted responses), encouragement (e.g. ‘Really?’ to keep participants engaged), elaboration (asking for more detail), clarification (seeking clarity), recapitulation (asking participants to restate or expand) and reflective (repeating participant phrases as questions to prompt deeper insight). These techniques helped elicit rich, detailed responses. The interview guide was piloted with three participants, one community manager and two community liaison officers to assess its effectiveness, and no changes were made after the pilot. All interviews were conducted in English. Participants had the option of having the interview done in isiZulu, but all participants preferred the English medium. Non-verbal cues were also noted in field notes, which helped during data analysis. The researcher used bracketing to reflect on personal biases and maintain transparency, and a reflective journal was kept throughout the study. The level of participation involved one-on-one interviews, which lasted between 31 min and 45 min, and member checks were performed on all transcripts to enhance the study’s rigour. The interview guide included 11 core open-ended questions, excluding recruiter characteristics and demographics. Additional prompts allowed participants to elaborate freely, consistent with a phenomenological, participant-led approach.

### Data analysis

Data collected in the study were analysed using an inductive thematic approach involving exploring and identifying recurrent patterns and themes. The data analysis was conducted using a flexible, iterative approach, where the researcher repeatedly reviewed, refined and adjusted the process to gain deeper insights rather than following a strict, step-by-step method (Baur 2019). Audio recordings were transcribed verbatim, capturing every word, including fillers. The researcher then reviewed the transcripts, highlighting keywords and phrases using open coding and assigned both constructed and in vivo codes. Relationships between coded transcripts, field notes and reflective notes were identified, and codes were grouped into preliminary themes. Sub-themes were organised into broader themes, which were refined to ensure clarity. The relevant data were transferred to Microsoft Excel, colour-coded and checked for overlap or missing themes before being organised into a table (Table 2). Finally, themes were named, and participant quotations were incorporated into the analysis, with the researcher reflecting on their influence throughout the process. All finalised themes and sub-themes were derived from participants’ lived experiences and analysed within the context of the facilities, communities and social environments where data were collected.

**TABLE 1:** Demographic and professional characteristics of recruiters involved in COVID-19 vaccine trials.

Category	Sub-category	Frequency	%
Gender	Male	8	57
	Female	6	43
Age groups (years)	20–29	2	14
	30–39	5	36
	40–49	5	36
	50–59	1	7
	60–69	1	7
Education	Grade 12	3	21
	Short courses	3	21
	Diploma	4	29
	Degree	4	29
Recruiting experience (Years)	0–5	7	50
	6–10	4	29
	More than 10 years	3	21
Number of COVID-19 vaccine trials recruited for	1	2	14
	2	5	36
	3	3	21
	4	4	29

COVID-19, coronavirus disease 2019.

**TABLE 2:** Summary of the themes and sub-themes that emerged from the data.

Themes	Sub-themes
1: Why people said yes – What drove participation	1.1 Financial motivations 1.2 Health and medical benefits 1.3 Fear of COVID-19 and personal loss 1.4 Prior Knowledge and trusted information 1.5 Social influence and community encouragement 1.6 Hope for protection against emerging strains 1.7 Recruiter engagement and trust-building
2: Barriers recruiters faced	2.1 Why people said no – Fears, mistrust, religion and daily struggles 2.2 Pressure and fear on recruiters in the field 2.3 COVID-19 restrictions made recruitment difficult
3: Recruiters’ strategies in the field	3.1 Collaboration with communities and key stakeholders 3.2 Making participation accessible 3.3 Community health empowerment through education 3.4 Involvement of various staff categories
4: Recruiters’ insights for improving future recruitment	4.1 Request for clear tools and guidance 4.2 More support builds trust 4.3 Staying visible and educating the community continuously 4.4 Leveraging social media to reach and inform

COVID-19, coronavirus disease 2019.

### Researcher characteristics and positionality: Ensuring scientific rigor and trustworthiness

The researcher’s public health background, insider-outsider position and reflective practices like journaling and bracketing helped minimise bias and provided valuable context for the study. To ensure scientific rigour, the study followed established trustworthiness criteria – using member checking, triangulation, audit trails and rich descriptions to enhance credibility, dependability, confirmability and transferability.

### Ethical considerations

Ethical clearance for this study was obtained from the University of KwaZulu-Natal Biomedical Research Ethics Committee (BREC) on 06 January 2023 (Reference No. BREC00004659/2022) and reviewed by the SAMRC Ethics Committee on 06 February 2023. Participants provided written informed consent, confirming they understood the study’s goals, procedures and any potential risks. Confidentiality was ensured by securely storing consent forms in a locked, fireproof cabinet, encrypting electronic data and pseudonymising identities (e.g., P1, P2, P3) on transcripts and recordings. Online interviews were conducted via Microsoft Teams in private settings, with only the researcher and participant present, ensuring privacy. The study complied with ethical guidelines established by the reviewing committees.

## Results

The 14 recruiters in this study represented a diverse group of individuals, many of whom had years of experience in participant recruitment for research. Most were in their 30s and 40s, with qualifications ranging from diplomas to degrees, and half had worked in recruitment for more than 6 years. Beyond these details, however, their lived experiences reveal the human realities of recruitment during a pandemic: resilience, pressure and emotion while navigating an extraordinary global health crisis. From their narratives, four central themes emerged, shedding light on what it meant to be on the frontlines of clinical trial recruitment during the COVID-19 pandemic: why people agreed to participate, the barriers recruiters encountered, the strategies they used to overcome those barriers and recruiters’ reflections and recommendations for the future.

### Theme 1: Why people said yes – What drove participation

#### Sub-theme 1.1: Financial motivations

For many participants, joining the COVID-19 vaccine trial was not just a choice – it was a necessity. Recruiters often saw how financial hardship pushed people to participate. P4 recalled one particularly moving moment:

‘We had a lady who was crying saying she’s unemployed. She was hoping that she’s going to get into the study because her neighbours, she can see them when they’re coming from their study, they going to buy food, you know.’ (Participant 4, Female, 48 years old)

In these communities, recruiters could see how deeply financial need influenced decisions, making participation feel like a lifeline.

#### Sub-theme 1.2: Health and medical benefits

Beyond money, access to healthcare motivated many participants. In areas with limited medical services, trial participation offered an opportunity for check-ups and guidance. P8 noted:

‘Clinical research sites they do things like medical check-ups and they; they even give family planning and do other tests. They want to keep their bodies healthy. They want to know everything about their bodies, that’s why they join.’ (Participant 8, Male, 34 years old)

Recruiters saw the relief and reassurance participants felt when they could monitor their health in a safe, structured way.

#### Sub-theme 1.3: Fear of coronavirus disease and personal loss

The reality of the pandemic was personal. Many had watched family or friends fall ill, and that fear often drove action. P14 reflected, ‘They saw that they are losing their loved ones. So, they saw the necessity or the need of vaccinating or taking those jabs’. Recruiters witnessed the pain behind these decisions, knowing that participants were often acting out of love and concern for their families.

#### Sub-theme 1.4: Prior knowledge and trusted information

Information empowered participants to make confident choices. Those who understood COVID-19 and vaccines, often through trusted sources, were more likely to participate. P9 shared, ‘People who accepted to participate in the study, who had full information about COVID-19, so they decided that they wanted to be a part of the trial’. Recruiters noticed that older healthcare workers especially valued guidance from doctors on television or radio, which helped them trust the process.

#### Sub-theme 1.5: Social influence and community encouragement

Community played a huge role. Participants often made decisions based on friends, neighbours or previous participants. P10 explained, ‘Those who, like were referred by participants who were exited with us on other studies. They will not take me where I will die’. And P11 observed, ‘Some of them doing it for the sake of making sure that the clinical trials are successful because they want to see the change’. Recruiters saw the power of peer influence and the shared hope of contributing to something bigger than themselves.

#### Sub-theme 1.6: Hope for protection against emerging strains

Many participants were forward-thinking, hoping the trial would give them protection against future COVID-19 variants. P2 remarked, ‘People, they want more vaccines for COVID because … we have different strains. So, if you have multiple vaccines, it won’t be easy if the COVID comes back again’. Recruiters could see how this hope offered participants a sense of control in uncertain times.

#### Sub-theme 1.7: Recruiter engagement and trust-building

Recruiters themselves were part of the solution. By sharing their experiences and being transparent, they built trust and eased fears. P1 explained, ‘You have to give your own information and show them your vaccination card and then they will believe that’s OK, they are eligible to the vaccine and participate in the study’. Through patience, empathy and genuine care, recruiters transformed the trial from a daunting process into a human connection.

While these motivators inspired many to participate, recruiters’ lived experiences also revealed the other side of the story: deep fears, resistance and difficulties that made enrolment far more challenging.

### Theme 2: What stood in the way – Barriers recruiters faced

#### Sub-theme 2.1: Why people said no – Fears, mistrust, religion and daily struggles

Recruiters faced numerous barriers as they tried to engage the public. Many individuals feared joining the trial because of potential side effects. P3 explained, ‘Vaccines having so many side effects and these vaccines got so many people sick from them’. Fear spread rapidly within communities, with people comparing themselves to others’ experiences before deciding to participate.

Distrust in recruiters and fear of needles were also common, as P11 shared, ‘I don’t trust you guys. In general, they don’t trust us. Well, these people, there’s a lot of frauds around here, you know, and many scams’. The recruiters believed that the community’s past experiences with scams and frauds fostered fear of being misled, as well as distrust of researchers and questioning of the intentions of clinical trials. Some individuals were reluctant to visit unfamiliar sites, with P9 noting, ‘Some of them were reluctant to take a taxi and come to a random place’.

Cultural, religious and family factors further shaped participation. Some declined vaccination because of cultural or religious beliefs. As P12 explained, ‘Another challenge is they show off their cultural and religious beliefs because when you approached him for vaccination, they were saying no because our culture doesn’t allow us to do so’. Family dynamics also influenced decisions, with some participants stopping communication because their partner was uncomfortable or because parents were involved in the decision-making process.

Misinformation, speculation and lack of knowledge created additional recruitment difficulties. False or inaccurate information often led individuals to make decisions based on incorrect beliefs. Rumours and misconceptions spread quickly. P6 described, ‘The community, most of the people in the community, talked about vaccines, which can kill you. The vaccine can cause a blood clot. The vaccine can do this. The vaccine can do that, and it’s hard to recruit them’. These fears and gaps in knowledge represented major barriers to recruitment.

Time constraints also hindered participation, as potential participants had limited availability because of work, family responsibilities, travel or other obligations. P13 explained:

‘They are extremely tired because they’ve been up the whole night working night shifts, so it was quite a challenge to an extent that we ended up not meeting them. Even with their lunch breaks, with the delay and clinic flow, if we couldn’t make a clinic flow fit in one hour, it was at least two hours.’ (Participant 13, Female, 47 years old)

Scheduling conflicts, long work hours and clinic delays made recruitment difficult.

#### Sub-theme 2.2: The pressure and fear on recruiters in the field

Recruiters navigated not only the recruitment difficulties in the community but also their own personal fears during a deadly pandemic. P2 admitted:

‘I was in fear of getting COVID-19. Then the thing that hit me very bad. It was my mom and my father – they had COVID at that time. I had to balance work and personal issues, so it wasn’t easy.’ (Participant 2, Female, 50 years old)

On top of personal stress, recruiters faced pressure from multiple simultaneous trials and competition from other research organisations for participants, adding further complexity to their work and the responsibility they carried in ensuring successful recruitment.

#### Sub-theme 2.3: When COVID-19 rules made recruitment difficult – Reaching participants safely

Pandemic restrictions made recruitment physically challenging. Face masks hindered communication, and some participants had difficulty hearing or understanding recruiters, while others did not wear masks. P10 observed, ‘There’s more than 10 people in their room and all of them they are not on mask’. Travel restrictions and limited site access created additional difficulties for recruitment. As P9 noticed, ‘You have to like acquire another permit to go to just to go to the next district’. Social distancing requirements reduced the number of participants who could travel together, and strict access policies made it difficult to reach certain populations, including older adults in care homes. Community gatherings were often prohibited, further limiting opportunities for engagement.

Despite these recruitment difficulties, recruiters did not give up. Their lived experiences also highlight persistence and creativity, as they drew on innovative strategies, teamwork and sheer determination to continue enrolling participants.

### Theme 3: Recruiters’ strategies in the field

#### Sub-theme 3.1 : Collaboration with communities and key stakeholders

Recruiters described how essential partnerships were in reaching potential participants. Recruiters worked closely with clinic staff, counsellors and peer educators, coordinating schedules and using local knowledge to reach communities. P1 explained, ‘Operation managers of the clinics will give us dates, and we will have meetings in maybe 1 week. Then, the following week … they provided us with a list’. Peer educators linked recruiters to participants, and social networks from previous trials helped identify others. Outreach also included childcare facilities and old-age homes, highlighting the importance of collaboration and trust.

#### Sub-theme 3.2 : Making participation accessible

Recruiters shared the lengths they went to make participation feasible for everyone, especially essential workers. P9 explained, ‘To accommodate everyone here to participate in the study, we had to implement the home visit for the clinics … We were recruiting essential workers, so we had to implement home visits’. Beyond visiting homes, they organised weekend clinics, offered transport support, distributed information through clinics and coordinated with nurses to identify eligible participants. Each step was carefully planned to reduce barriers and make participation as convenient as possible.

#### Sub-theme 3.3: Community health empowerment through education

Education was at the heart of recruiters’ strategies. They recognised that providing clear, accurate information was essential to empower communities to make informed decisions. P9 shared, ‘To have education sessions at the healthcare clinics in the morning and afternoon … we had an open discussion to educate them about COVID-19 and the research organisation’. Recruiters conducted structured sessions, addressed questions openly and engaged in dialogue with participants to build understanding and trust, showing how knowledge could directly influence willingness to participate.

#### Sub-theme 3.4 : Involvement of various staff categories

Recruiters stressed that recruitment was never an individual effort. P9 stated, ‘Everyone at the site was involved in recruitment at this time because we needed help’. Supporting each other helped reach more participants and highlighted the importance of teamwork.

Beyond immediate strategies, recruiters also reflected deeply on their work. Their lived experiences during COVID-19 pandemic shaped important recommendations for how recruitment could be strengthened in future trials and public health emergencies.

### Theme 4: Recruiters’ insights for improving future recruitment

#### Sub-theme 4.1: Request for clear tools and guidance

Recruiters found it challenging to engage communities when tools were unclear or incomplete, often answering questions that study pamphlets could not. P11 said, ‘Study pamphlets handed out to the community are too vague and need more information about the study’. They noticed the protocol was too scientific, and simpler procedures, clearer materials and more preparation time would have helped them connect meaningfully with participants. It was not just about following a protocol – it was about making the study understandable for those new to clinical research.

#### Sub-theme 4.2: More support builds trust

Recruiters require strong staff support to succeed. They face moments when participants ask complex questions or feel anxious, and the pressure can be overwhelming. P14 emphasised this need:

‘Basically, it’s all about piece of information, empowering the staff first and making sure that we have well, capacitated staff who will deliver the right message to the people who will attend to all the questions in the most uninformed way.’ (Participant 14, Male, 29 years old)

In the future, recruiters will also require additional staff, vehicles and branded uniforms to manage anxious participants, handle heavy workloads and maintain trust and visibility in the community.

#### Sub-theme 4.3: Staying visible and educating the community continuously

Recruiters also reflected on the power of visibility and consistent engagement. They described how trust was built slowly, not in a single visit, but through repeated presence, conversations and educational sessions. P10 explained, ‘We need to improve … to show that you are part of them, your part of the community, visible in the community, engaging the leadership … include everyone, not desert anyone’.

#### Sub-theme 4.4: Leveraging social media to reach and inform

Finally, recruiters recognised that their efforts could be amplified through social media. They saw platforms such as Facebook not just as a tool but as a bridge to reach people who might never attend a clinic or community meeting. P4 reflected, ‘The Facebook page is where we will start … people will get the correct information’.

## Discussion

This study explored the lived experiences of recruiters involved in COVID-19 vaccine trials in South Africa, highlighting the complex interplay of community motivations, barriers and recruitment strategies during a time of global uncertainty. Recruitment was not simply a technical process but an emotionally demanding practice for recruiters, who had to navigate participants’ financial hardship, health needs, social influences and misinformation, while also contending with pandemic restrictions themselves. These findings extend existing knowledge by centring the voices of recruiters, whose perspectives are often overlooked in clinical trial research.

The findings reveal that participants’ decisions to join COVID-19 vaccine trials were influenced by a combination of economic, emotional, informational and social factors, highlighting the complex motivations that shaped enrolment in this context.

Economic hardship was a powerful driver of participation, with many individuals viewing reimbursement as financial relief particularly during a period of widespread job losses and financial insecurity. As reported in a South African study, many unemployed individuals viewed reimbursement as financial relief (Nkosi, Mulopo & Schmidt 2024). These motivations were heightened by widespread job losses and financial insecurity during the pandemic (Simon & Khambule 2022). Access to healthcare was also a motivator, consistent with findings from a survey on willingness to participate in COVID-19 vaccine trials among healthcare workers in Uganda (Kitonsa et al. 2021).

Emotional motivations also played a central role, particularly the desire to protect loved ones. Research in behavioural science has shown that people often make health decisions with the well-being of others in mind (Sommers et al. 2025). A cross-sectional survey in Egypt, Saudi Arabia and Jordan found that 80.5% of participants were motivated to join trials to protect their families (Abdelhafiz et al. 2021). Similarly, participants in a South African study on the perceptions, experiences and motivations of COVID-19 vaccine trial participants reported that their decisions to join COVID-19 vaccine trials were influenced by a sense of responsibility for protecting their family’s well-being, with many viewing vaccination as a way to safeguard loved ones (Nkosi et al. 2024). Future research should examine how grief, fear and perceptions of vaccine effectiveness shape both hesitancy and participation.

Knowledge and trusted information also influenced willingness to enrol. In India, knowledge of vaccine development positively shaped enrolment decisions (Goel et al. 2022). However, in low- and middle-income countries, knowledge alone does not always increase willingness to participate in clinical trials; trust, perceived benefits and safety concerns also play a crucial role (Abdelhafiz et al. 2021). Behavioural science highlights that information provision is rarely sufficient; social persuasion and trusted figures are often required to counter negative influences, including religious concerns (Fishbein & Ajzen 2011; Michie, Van Stralen & West 2011).

The COVID-19 pandemic was unprecedented, with vaccines developed rapidly amid limited knowledge of transmission, immunity and prevention (Bavel et al. 2020). Evolving scientific guidance and frequent updates, amplified via social media, created confusion, reduced compliance and fuelled misinformation and conspiracy theories (Dean et al. 2023; Lu et al. 2021; Mohammed et al. 2022). Future health crises require clear, consistent communication, improved health literacy, proactive misinformation management, engagement of trusted community leaders and transparency in scientific processes to strengthen public trust in vaccines and health measures.

Social influence also emerged as a strong motivator. Encouragement from peers and previous participants shaped decisions, underscoring the value of referral programmes such as Pfizer’s structured incentive approach (Pfizer 2023). Some participants joined trials to contribute to science itself, supporting findings from Saudi Arabia where scientific contribution was the most significant motivator (Felemban, Tashkandi & Mohorjy 2021).

Trust was central to recruitment. Participants directly requested to see recruiters’ vaccination certificates, reinforcing the importance of transparency (Latkin et al. 2021). However, these insights are based only on recruiters’ perspectives. The effectiveness of such strategies may depend on recruiters’ perceived relationship with communities; being seen as outsiders could undermine credibility. To strengthen evidence, future studies should capture participants’ perspectives and examine whether strategies such as disclosing vaccination status are effective across cultural and socioeconomic settings. While these motivators helped people say yes, recruiters also encountered significant obstacles in the field. These barriers were diverse, spanning psychological fears, social pressures, misinformation and logistical difficulties, which together shaped the recruitment experience in complex ways.

Recruiters described multiple barriers that hindered participant enrolment in COVID-19 vaccine trials. Among the most prominent were fear, misinformation, cultural and religious beliefs, logistical difficulties and time constraints. These reflections resonate with broader patterns of vaccine hesitancy observed during the pandemic, where rapidly evolving information, social myths and uncertainty undermined public confidence (Dean et al. 2023; Mohammed et al. 2022). Recruiters emphasised that successful engagement required not only addressing practical obstacles but also responding empathetically to participants’ emotional concerns, through clear communication and reassurance from trusted figures.

A pervasive barrier reported by recruiters was participants’ fear of the unknown and apprehension about vaccine side effects. These fears echo findings from prior research highlighting psychological factors as significant obstacles to trial participation (Abu-Farha, Alzoubi & Khabour 2020; Sun et al. 2021; Yuh et al. 2022). The Health Belief Model (Maiman & Becker 1974) underscores how perceived susceptibility, severity and risk concerns shape health behaviours (Maiman & Becker 1974). Recruiters observed that the rapid development and media attention surrounding COVID-19 vaccines heightened these fears, reinforcing hesitancy.

Mistrust in recruiters and fear of needles also limited enrolment. Recruiters reported that participants often questioned their intentions or expertise, making rapport-building essential. As literature suggests, trust in the credibility of recruiters strongly influences willingness to participate (Latkin et al. 2021). Recruiters found that peer encouragement, education and reassurance were critical strategies for overcoming these barriers.

Logistical concerns, such as reluctance to travel to unfamiliar sites, further impeded recruitment. Participants expressed anxiety about visiting clinical sites for the first time. This aligns with the Theory of Planned Behavior, which posits that perceived behavioural control – shaped by social and practical constraints – influences actions (Ajzen 1991). Recruiters highlighted the importance of offering accessible study locations or mobile clinics to reduce travel-related barriers.

Cultural and religious norms, alongside family and partner influence, played a significant role in participants’ decisions. Recruiters frequently encountered situations where family objections or religious beliefs limited engagement. As reported in the literature, social norms and familial expectations strongly shape health behaviours (Ni, Chen & De Brujin 2021). Recruiters may benefit from collaborating with religious leaders and involving trusted family members to foster culturally sensitive approaches and potentially improve participation.

Limited awareness of COVID-19 and the vaccine trial itself, particularly in rural areas, presented another obstacle. Recruiters observed that participants often misunderstood study objectives or held inaccurate beliefs about vaccines. Framing theory suggests that the way information is presented significantly affects interpretation (Entman 1993). Recruiters emphasised that clear, consistent messaging delivered via trusted community figures improved understanding and countered misinformation (Dhai 2021; Nguyen et al. 2022).

Time constraints were also frequently cited. Participants, especially healthcare workers and individuals with demanding schedules, struggled to commit to trial visits. Recruiters reported that offering flexible scheduling, transport support and clear communication about time commitments was essential to mitigate this barrier.

Recruiters themselves faced personal fears and pressures while conducting fieldwork. Many expressed concerns for their own safety and the wellbeing of family members, echoing literature on occupational stress among frontline workers during the pandemic (Chair et al. 2023; Elaidy et al. 2023). Balancing personal concerns with the demands of recruitment created emotional strain, highlighting the need for mental health support and wellness programmes to maintain recruiter effectiveness.

Finally, COVID-19 restrictions, including social distancing, mask mandates and travel limitations complicated recruitment. Recruiters described difficulties communicating through masks, maintaining confidentiality and accessing restricted facilities such as old-age homes. These operational difficulties reflect broader concerns about public health measures affecting social interactions (Lehmann & Lehmann 2021). Recruiters found that mobile units, remote study visits and community-based strategies were effective in reaching participants safely while reducing exposure risk.

Overall, recruiters’ experiences illustrate that barriers to vaccine trial participation were multifaceted, encompassing psychological (fear, mistrust), social (family, religion), informational (knowledge gaps, misinformation) and operational (time, travel, site access) dimensions. These barriers align with the broader literature (Abu-Farha et al. 2020; Dhai 2021; Latkin et al. 2021; Sun et al. 2021).

Addressing these recruitment difficulties requires flexible, culturally sensitive recruitment strategies that combine transparent communication, peer and community engagement, logistical support and mental health resources for recruiters. Understanding and mitigating these barriers is critical for ensuring timely and effective vaccine trial recruitment.

Recruiters employed a variety of strategies to enhance participant recruitment in COVID-19 vaccine trials, reflecting the need for flexible, culturally sensitive and community-centred approaches. Collaboration with stakeholders and communities emerged as a central strategy. Recruiters worked closely with healthcare professionals, old-age homes, peer educators, community working groups and community advisory boards to build trust and facilitate access to diverse populations. This aligns with prior research showing that partnerships with trusted community members and healthcare personnel improve recruitment and enrolment in clinical trials (Castellon-Lopez et al. 2023; Folayan et al. 2021; Wilkinson 2020). For example, the Novavax trial demonstrated that sustained community engagement fosters a more diverse volunteer register, emphasising the importance of ongoing partnerships (Andrasik et al. 2021). Peer educators, in particular, served as bridges between researchers and the community, enhancing participants’ understanding and trust, which this study confirms as a critical mechanism in COVID-19 vaccine trials.

Targeting hard-to-reach populations was another key strategy. Recruiters engaged old-age homes to increase enrolment among older adults, a group frequently underrepresented in clinical trials and facing accessibility difficulties during pandemics (Arevalo et al. 2016; Kim et al. 2021). Involving community working groups and community advisory boards helped researchers to understand community concerns, ensure cultural appropriateness and promote ethical recruitment (Folayan et al. 2021). By leveraging local voices, recruiters could facilitate informed consent and encourage participation among populations that might otherwise be hesitant, reinforcing the value of community engagement in overcoming structural and social barriers.

Making participation accessible was a consistent theme. Strategies such as extending recruitment catchment areas, conducting door-to-door recruitment, arranging home visits, organising weekend clinics, providing transport support and distributing informational pamphlets reflect a participant-centred approach. Literature supports these flexible measures as effective in mitigating logistical and time-related barriers (Arevalo et al. 2016; Karasik 2022; Newington & Metcalfe 2014; Valerio et al. 2016; Winifred Ekezie et al. 2020). By reducing the burden of participation, these approaches promote inclusivity and mitigate inequalities in trial enrolment, which is especially critical during a public health emergency.

Community health empowerment through education was also emphasised. Recruiters provided educational sessions in clinics and community spaces, improving participants’ understanding of trial objectives and relevance to personal and public health. This mirrors findings that informed communities are more likely to engage in research (Winifred Ekezie et al. 2020). Educating gatekeepers and sharing accurate COVID-19 data strengthened trust, reduced misinformation and promoted engagement, demonstrating that empowerment through education enhances community ownership of the research process.

Finally, the involvement of multiple staff categories facilitated rapid and efficient enrolment. Interdisciplinary collaboration and flexible staff roles are widely recognised as critical to overcoming recruitment difficulties and meeting time-sensitive targets in COVID-19 research (Helmer et al. 2021). This study highlights how team-based recruitment enabled knowledge sharing, optimised resource use and maintained momentum in a high-pressure environment.

In synthesis, recruitment strategies for COVID-19 vaccine trials were multifaceted, combining stakeholder collaboration, accessibility, education and team-based approaches. These strategies align with global evidence while extending understanding to pandemic-specific contexts, particularly regarding peer educators and engagement with vulnerable populations. Collectively, such approaches foster trust, enhance informed participation and support the timely achievement of enrolment targets, underscoring the importance of adaptive, community-oriented recruitment practices during health emergencies. These lessons from the field lead to recruiters’ reflections on how recruitment could be improved in future trials.

Recruiters reflected on ways to improve future trial recruitment, emphasising the need to support and equip recruiters. Providing simplified trial protocols and clear pamphlets helps recruiters understand and explain the study confidently to participants. This aligns with studies showing that when recruiters fully grasp protocols, they can answer questions, build trust and improve recruitment outcomes (Al-JunDi & SAkkA 2016; Gardner, Treweek & Gillies 2019; Winifred Ekezie et al. 2020). Recruiters also highlighted the importance of having preparation time before the trial begins, especially during pandemics when recruitment timelines are short (Meskell et al. 2022). Well-prepared recruiters can engage participants more effectively and ensure higher-quality enrolment.

Optimising study infrastructure was another key recommendation. Training, mentorship and capacity building help recruiters feel confident and motivated, which improves their performance (Mbangeleli & Ojugbele 2021; Modise 2023; Taris & Schaufeli 2015). Recruiters also suggested providing visible identification, branded uniforms and protective equipment. Although often overlooked, these simple measures can increase professionalism, visibility and safety, making participants more likely to trust and engage with the study.

Recruiters stressed the value of continuous community engagement and education. Building trust requires understanding community-specific concerns, cultural beliefs and motivations (Castellon-Lopez et al. 2023; Vangeepuram et al. 2023). Ongoing engagement allows recruiters to address fears and misinformation and to involve communities in the research process. Education sessions, clear explanations and sharing accurate COVID-19 information empower participants, helping them make informed decisions and increasing participation, consistent with global evidence on effective trial recruitment.

Using social media was identified as a practical tool for reaching more participants, especially when in-person access is limited during pandemics. Digital platforms allow recruiters to target diverse populations and quickly share accurate information, which is important for vaccine safety and efficacy evaluation (Darmawan et al. 2020; Wisk & Buhr 2021). As the framing theory suggests, effectively framing messages can reduce misinformation and encourage participation (Entman 1993). Training recruiters to use social media ethically and sensitively ensures maximum benefit and minimises risks.

Overall, recruiters’ insights show that improving recruitment requires a multilayered approach: supporting and training recruiters, optimising infrastructure, engaging and educating communities continuously and using digital tools wisely. These strategies directly address barriers such as fear, mistrust, logistical difficulties and misinformation. Linking these recommendations to established frameworks, such as the health belief model, theory of planned behaviour and framing theory, provides a clear path to enhance participant trust, diversity and recruitment success. Implementing these measures can make future vaccine trials more efficient, inclusive and community-centred.

### Strengths and limitations of the study

A key strength of this phenomenological study is its focus on the lived experiences of community recruiters in COVID-19 vaccine clinical trials. By using open-ended interviews, recruiters could share rich, detailed narratives about difficulties they faced, including community mistrust, fear and personal risk. These insights captured nuances that quantitative methods might miss. In addition, providing recruiters with a platform to voice their experiences empowered them to highlight their needs and advocate for better support systems, offering valuable guidance for improving recruitment strategies in future clinical trials.

A limitation is the researcher’s insider status, which could influence data interpretation. Although familiar with the broader organisational context, the researcher lacked specific knowledge of participants’ roles and subculture. The researcher maintained reflexivity through reflective journaling, repeated transcript coding and post-interview reflections. Member checks were conducted to validate participants’ statements, ensuring accurate and authentic representation of their lived experiences while minimising potential bias.

### Recommendations

Recommendations based on recruiters’ lived experiences highlight the importance of incorporating their perspectives into clinical trial planning and execution. Continuous dialogue with recruiters allows recruitment difficulties to be addressed promptly, fostering trust, improving participation and enhancing overall trial success. Actively involving recruiters in creating study materials, planning community outreach and delivering education initiatives ensures recruitment is culturally sensitive, understandable and trusted. Their insights guide the process, while community involvement helps participants better understand the study. Lastly, prioritising mental health support and infection-prevention training safeguards recruiters’ well-being and sustains motivation under challenging conditions.

## Conclusion

This study shed light on the multifaceted experiences of community recruiters and the complex landscape of participant recruitment for COVID-19 vaccine clinical trials during a pandemic in the eThekwini District, KZN. Understanding the facilitators and barriers from the recruiters’ perspectives could contribute to developing effective and inclusive recruitment strategies and more robust clinical research recruitment planning in the future. The strategies recruiters implemented and their recommendations offered valuable insights for improving future recruitment efforts. It is essential to build on these insights to develop robust recruitment practices that can respond effectively to global health crises.
